# Coexistence of Elevated Mean Platelet Volume and Bleeding Symptoms in Chronic Kidney Disease: A Discordant Hemostatic Profile

**DOI:** 10.7759/cureus.111383

**Published:** 2026-06-23

**Authors:** Ayomide Adeyeye, Tehzeeb Abdul Sattar, Kaushalendra Mani Tripathi, Sahil Ali, Paul Sunday Samuel, GKM Rashik Uzzaman, Ayodeji Oso, Mahmoud AM Abughazal, Sibgha Shreen

**Affiliations:** 1 Medicine, Zaporizhzhia State Medical and Pharmaceutical University, Zaporizhzhia, UKR; 2 Medicine, East and North Hertfordshire NHS Trust, Stevenage, GBR; 3 Pathology, Liaquat University of Medical and Health Sciences, Jamshoro, PAK; 4 Internal Medicine, White River Health, Batesville, USA; 5 Medicine, Chandka Medical College, Larkana, PAK; 6 Geriatrics, Abia State University, Uturu, NGA; 7 Geriatrics, Barking, Havering and Redbridge University Hospitals NHS Trust, Ilford, GBR; 8 Stroke Medicine, United Lincolnshire Teaching Hospitals NHS Trust, Lincoln, GBR; 9 General Medicine, Royal Shrewsbury Hospital, Shrewsbury, GBR; 10 Medicine, Peoples University of Medical and Health Sciences for Women, Nawabshah, PAK; 11 Medicine, University Hospitals Birmingham NHS Foundation Trust, Birmingham, GBR

**Keywords:** bleeding symptoms, chronic kidney disease, hemostasis, mean platelet volume, platelet indices

## Abstract

Background: Hemostasis in chronic kidney disease (CKD) is currently becoming a complicated and disturbed process, not a simple bleeding disorder. Recent evidence suggests that the combination of elevated mean platelet volume (MPV) and bleeding, particularly during the initial phases of the disease, is a discordant hemostatic profile.

Objective: To examine the association between mean platelet volume (MPV) and bleeding symptoms among patients with chronic kidney disease (CKD) and to evaluate the utility of platelet indices in characterizing a discordant hemostatic profile.

Methods: This analytical cross-sectional study recruited 527 patients with CKD from tertiary care centers. Bleeding was measured on a questionnaire that included typical bleeding symptoms and a bleeding score. Platelet indices, such as mean platelet volume (MPV), platelet distribution width (PDW), and platelet count, were provided in the medical records. Data were evaluated using nonparametric tests, logistic regression, and receiver operating characteristic (ROC) curves.

Results: Bleeding symptoms were observed in 298 patients (56.5%). Bleeding participants had increased MPV, which increased with increasing CKD stages. Multivariate analysis showed MPV was significantly associated with bleeding symptoms (OR = 2.88, p < 0.001). ROC curves indicated moderate discriminative ability (area under the curve (AUC) = 0.730), but poor performance of PDW and non-predictive value of platelet count.

Conclusion: CKD is associated with a discordant hemostatic profile of elevated MPV alongside bleeding symptoms. MPV may serve as a potential marker associated with bleeding symptoms in patients with CKD.

## Introduction

Hemostasis in chronic kidney disease (CKD) is beginning to be viewed as a complicated and dysregulated process as opposed to a simple imbalance between bleeding and thrombosis [1]. Historically, CKD was associated with a tendency to bleed, largely explained by platelet dysfunction caused by uremia and platelet-vessel wall interactions [2,3]. However, recent evidence shows that, in patients with CKD, platelet size and activity can be increased simultaneously in the early stages of the disease, thereby contributing to the prothrombotic state [4,5]. This paradox indicates that the conventional understanding of hemostatic alterations in CKD is mostly opposite and results in a more complex and diverse phenotype [1].

The presence of altered platelet activity and manifestation of bleeding symptoms cannot be fully attributed to traditional patterns of platelet dysfunction [6,7]. Even though enhanced platelet activation may increase thrombotic risk, qualitative defects in platelet adhesion, aggregation stability, or endothelial interactions that also predispose people to bleeding can occur [8]. In addition, uremic toxins might selectively influence various elements of hemostasis, with both stimulatory and inhibitory influences on platelet functions resulting in a state of functional discordance [1]. These findings indicate that the majority of available studies have examined predisposition to either bleeding or thrombotic processes, but not both, particularly in early CKD [9,10].

The clinical significance of this dual hemostatic behavior is that it directly affects the risk stratification and therapeutic decision-making [9,11]. Early CKD can be considered a critical phase in which platelet activity can be modified, resulting in cardiovascular problems in the future [5]. Therefore, the objective of the present study was to examine the association between mean platelet volume (MPV) and bleeding symptoms among patients with chronic kidney disease (CKD) across stages I-V and to evaluate the utility of platelet indices in characterizing a discordant hemostatic profile marked by the coexistence of elevated platelet activity and bleeding manifestations. By doing so, the research will contribute to the overall knowledge of hemostatic dysregulation in CKD and inform future research and practice.

## Materials and methods

Study framework and setting

This cross-sectional analytical study aimed to investigate hemostatic variation in patients with chronic kidney disease (CKD) with a specific emphasis on determining a discordant hemostatic profile. This clinical picture was theorized as coexisting elevated MPV and bleeding manifestations, reflecting a condition of hemostatic discordance, but not a solitary dysfunction. The study was conducted in Watim General Hospital, Rawalpindi, Pakistan, where both clinical and laboratory testing were available from December 2025 to March 2026. A mixed-methods design that combines questionnaire-based evaluation and laboratory data extraction was adopted to provide a comprehensive assessment of both subjective and objective factors of hemostasis.

Participant selection and characteristics

The sample size was recruited in inpatient and outpatient departments within the study period. To include adult patients, CKD patients who were clinically diagnosed were approached. The demographic and clinical data, including age, gender, body mass index (BMI), smoking, hypertension, diabetes mellitus, and CKD duration and medication history, were collected using a structured pro forma.

These variables were used to characterize the study population and to control for known confounding variables that influence platelet function and bleeding risk. Consecutive eligible patients presenting to the inpatient and outpatient departments during the study period were approached for participation. Patients who met the inclusion criteria and provided informed consent were enrolled in the study.

Sampling strategy and sample size determination

The researchers used non-probability convenience sampling to select the participants, i.e., all qualified patients who reported during the study were enrolled. This method was selected because it is practical in a clinical setting, and the study was exploratory. The final sample size of 527 was considered adequate to estimate the prevalence of high MPV and bleeding symptoms and to approximate these prevalences among the study participants.

The standard assumptions of cross-sectional studies were used to determine the sample size, which was based on the variability in platelet dysfunction and bleeding phenotypes previously reported in CKD populations. The lack of precise local estimates necessitated using a conservative prevalence estimate to ensure adequate statistical power and representation [12]. The final sample also accounted for incomplete responses or missing data. Inclusion and exclusion criteria are summarized in Table 1.

**Table 1:** Participant Eligibility Criteria eGFR = Estimated Glomerular Filtration Rate. This summarizes the inclusion and exclusion criteria used to select participants.

Category	Criteria
Inclusion Criteria	Adult patients (18 years and above) with a known diagnosis of chronic kidney disease (CKD), as defined by clinical assessment and laboratory values (e.g., serum creatinine and eGFR)
Exclusion Criteria	Known hematological disorders
-	Active infections
-	Malignancies
-	Chronic inflammatory conditions

Demographic and clinical assessment

The selected questions in the demographic and clinical proforma were used to assess bleeding symptoms. These products contained symptoms such as epistaxis, bleeding gums (particularly during brushing), bruising, bleeding persisting for more than five minutes following minor cuts, and menorrhagia (in women). This was a symptom-based screen, as the evaluation was based on self-reported symptoms rather than a formal bleeding assessment tool. The questionnaire was administered through face-to-face interviews by trained study personnel. The instrument was developed specifically for this study and included questions regarding epistaxis, gum bleeding, easy bruising, prolonged bleeding following minor cuts, and menorrhagia in female participants. Responses were recorded as present or absent based on participant self-report.

Analysis was performed using two measures. First, a dichotomous variable (bleeding symptoms: Yes/No) was constructed, where Yes implied the reporting of at least one symptom. Second, a symptom score (0-5) was calculated based on the number of symptoms reported. With this, there were categories: no symptoms (0), mild symptoms (1-2), and severe symptoms (>3). This dual approach allowed both simplified outcome assessment and a more nuanced evaluation of bleeding burden (Appendix 1).

Laboratory evaluation of platelet activity

Laboratory data, such as major platelet indices and renal functional parameters, were available in the patient's medical records. The primary measure of platelet activity was the mean platelet volume (MPV), as it was considered a surrogate measure of platelet size and activity. Supportive indices included platelet distribution width (PDW) and platelet count, which provided further insight into platelet morphology and variability.

Renal function was assessed using serum creatinine and the estimated glomerular filtration rate (eGFR), which were then used to classify the CKD stage. CKD stages were classified according to Kidney Disease: Improving Global Outcomes (KDIGO) eGFR categories as follows: Stage I (≥90 mL/min/1.73 m²), Stage II (60-89), Stage III (30-59), Stage IV (15-29), and Stage V (<15) [13]. All laboratory values were reported in accordance with the standardized reporting guidelines of the relevant institutions, thereby introducing consistency and reliability in measurements.

Definition of hemostatic outcomes

The primary outcome of interest was a discordant hemostatic profile, with a high mean platelet volume (MPV) and bleeding symptoms. The variable of interest was bleeding symptoms (Yes/No). The independent variable was selected as MPV, and the auxiliary indicators of platelet activity were selected as PDW and platelet count. Covariates considered the possible confounding factors, such as age, gender, smoking status, diabetes mellitus, hypertension, and CKD stage. The design enabled the evaluation of the independent and interactive effects of platelet activity on the bleeding outcomes.

Statistical analysis plan

Data were entered and analyzed in IBM SPSS Statistics version 26 (IBM Corp., Armonk, New York, USA). The demographic, clinical, and laboratory variables were described using descriptive statistics, with categorical variables presented as frequencies and percentages and continuous variables as means and standard deviations. The Kolmogorov-Smirnov test was used to assess the normality of the continuous variables, which showed they were non-normal; therefore, non-parametric tests were used.

Although the MPV was normally distributed, nonparametric tests were applied throughout the analysis since the other variables were not normally distributed. Spearman's rank correlation analysis was used to determine the relationships between platelet indices and bleeding outcomes. Binary logistic regression analysis was used to identify independent predictors of bleeding symptoms, with results reported as odds ratios (ORs) and 95% confidence intervals (CIs). Medication use (aspirin, clopidogrel, anticoagulants, and non-steroidal anti-inflammatory drugs (NSAIDs)) was recorded descriptively but was not included in the multivariable regression model because the primary objective was to evaluate the association between platelet indices and bleeding symptoms. The potential confounding effect of these medications is acknowledged as a study limitation. Receiver operating characteristic (ROC) curves were used to analyze mean platelet volume (MPV) and assess its discriminative power. A p-value less than 0.05 was considered significant. No missing data were identified for the variables included in the final analysis; therefore, complete-case analysis was performed.

Ethical considerations

This study received ethical approval from the relevant Institutional Review Board (IRB) of Watim Medical College Rawat, Rawalpindi, Pakistan (approval no.: WMCR-IRB-03C1-2025, dated: 25-11-2025), and data collection was subsequently conducted. The study was conducted in accordance with the stipulated ethical guidelines for research involving people, including respecting their autonomy and confidentiality and ensuring the confidentiality of data. All participants gave informed written consent before enrolling. The participants were adequately informed about the purpose of the research, the nature of the data to be collected, and that they could leave the study at any time without consequences.

All data were anonymous, and no personally identifiable information was collected. The data were used exclusively for research, and information about participants was considered confidential. Data security and integrity were ensured because only the research team was allowed to access the data.

## Results

Table 2 shows participant characteristics of 527 individuals who participated in the study. The majority of participants were in the 56-65 age range (127 individuals, 24.1%) and the 46-55 age range (125 individuals, 23.7%). The younger age group (18-30 years) had 67 participants, representing 12.7% of the total. Males (n = 300, 56.9%) outnumbered females (n = 227, 43.1%). Most participants had normal BMI (n = 179, 34.0%) or were overweight (n = 160, 30.4%). A large proportion were non-smokers (n = 230, 43.6%), while hypertension (n = 288, 54.6%) and diabetes (n = 238, 45.2%) were common. The most common duration for CKD was one to three years, affecting 177 patients (33.6%), and Stage III CKD was the most common form of the illness, occurring in 196 patients (37.2%). The study participants used two medications: aspirin (n = 169, 32.1%) and clopidogrel (n = 99, 18.8%). The study found that 106 participants (20.1%) experienced easy bruising, while 98 participants (18.6%) suffered from epistaxis. The study found that more than half of the participants, 298 out of 527 (56.5%), showed signs of bleeding.

**Table 2:** Demographic and Clinical Characteristics of Study Participants (N = 527) CKD = Chronic Kidney Disease; BMI = Body Mass Index; NSAIDs = Non-Steroidal Anti-Inflammatory Drugs. Menorrhagia was recorded for females only (n = 227).

Variable	n	%
Age Group		
18-30 years	67	12.7
31-45 years	91	17.3
46-55 years	125	23.7
56-65 years	127	24.1
Above 65 years	117	22.2
Gender		
Male	300	56.9
Female	227	43.1
BMI Category		
Underweight (<18.5)	78	14.8
Normal (18.5-24.9)	179	34.0
Overweight (25-29.9)	160	30.4
Obese (≥30)	110	20.9
Smoking Status		
Never Smoked	230	43.6
Ex-Smoker	162	30.7
Current Smoker	135	25.6
Hypertension		
Yes	288	54.6
No	239	45.4
Diabetes Mellitus		
Yes	238	45.2
No	289	54.8
CKD Duration		
Less than 1 year	151	28.7
1-3 years	177	33.6
3-5 years	126	23.9
More than 5 years	73	13.9
CKD Stage		
Stage I	55	10.4
Stage II	100	19.0
Stage III	196	37.2
Stage IV	119	22.6
Stage V	57	10.8
Medication Use		
Aspirin	169	32.1
Clopidogrel	99	18.8
Anticoagulants	72	13.7
NSAIDs	86	16.3
Bleeding Symptoms		
Epistaxis	98	18.6
Gum Bleeding	79	15.0
Easy Bruising	106	20.1
Prolonged Bleeding	89	16.9
Menorrhagia	34	6.5
Bleeding Symptoms (Overall)		
Present	298	56.5
Absent	229	43.5

Table 3 presents descriptive statistics and normality analyses for continuous variables (N = 527). The mean MPV was 9.35 ± 1.00 fL (range: 7.00-12.67) and was the only variable with a normal distribution (Kolmogorov-Smirnov (K-S) p = 0.200; Shapiro-Wilk (S-W) p = 0.098). In contrast, PDW had a mean of 10.33 ± 1.37% (range: 9.00-15.50) and showed significant deviation from normality (K-S p < 0.001; S-W p < 0.001) with positive skewness (0.989). Platelet count (253.35 ± 55.96 ×10⁹/L), serum creatinine (2.62 ± 0.89 mg/dL), and eGFR (48.84 ± 23.67 mL/min/1.73m²) also demonstrated non-normal distributions, as supported by significant Shapiro-Wilk p-values (< 0.001). In summary, except for MPV, none of the variables followed a normal distribution, and thus, nonparametric tests are warranted.

**Table 3:** Descriptive Statistics and Tests of Normality for Continuous Variables (N = 527) M = Mean; SD = Standard Deviation; Min = Minimum; Max = Maximum; K-S = Kolmogorov-Smirnov; S-W = Shapiro-Wilk; MPV = Mean Platelet Volume; PDW = Platelet Distribution Width; eGFR = Estimated Glomerular Filtration Rate. MPV was the only variable that satisfied the normality criteria for both tests. All remaining variables demonstrated significant deviation from normality, justifying the use of nonparametric tests.

Variable	M	SD	Min	Max	K-S Statistic	K-S p	S-W Statistic	S-W p	Skewness	Kurtosis
MPV (fL)	9.35	1.00	7.00	12.67	0.033	0.200	0.995	0.098	0.132	0.103
PDW (%)	10.33	1.37	9.00	15.50	0.164	<0.001	0.876	<0.001	0.989	0.388
Platelet Count (×10⁹/L)	253.35	55.96	150	450	0.034	0.192	0.988	<0.001	0.260	-0.210
Serum Creatinine (mg/dL)	2.62	0.89	0.80	4.95	0.034	0.200	0.989	<0.001	0.026	-0.0698
eGFR (mL/min/1.73 m²)	48.84	23.67	15.0	90.0	0.076	<0.001	0.941	<0.001	0.204	-1.016

Table 4 shows the comparison of MPV between participants with and without bleeding symptoms (N = 527) using the Mann-Whitney U test. Patients with bleeding symptoms (n = 298) had a markedly higher mean rank (316.58; sum of ranks = 94,341.50) compared to those without bleeding (n = 229; mean rank = 195.57; sum of ranks = 44,786.50). Patients with bleeding symptoms had significantly higher MPV values compared to those without symptoms, resulting in a statistically significant difference between the two groups (U = 18,451.50, Z = -9.043, p < 0.001).

**Table 4:** Mann-Whitney U Test Comparing MPV Between Bleeding Symptoms Groups (N = 527) MPV = Mean Platelet Volume. The Mann-Whitney U test was used because the grouping variable was non-normally distributed. A significantly higher mean MPV rank was observed in patients with bleeding symptoms compared with those without, indicating elevated MPV in the bleeding group.

Group	n	MPV (fL), Median (Q1-Q3)	Mean Rank	Sum of Ranks	U	Z	p
No Bleeding Symptoms	229	8.94 (8.34-9.43)	195.57	44,786.50	18,451.50	-9.043	<0.001
Bleeding Symptoms Present	298	9.62 (9.12-10.33)	316.58	94,341.50	-	-	-

The Kruskal-Wallis test was used to compare MPV measurements across different CKD stages in a study with 527 participants, as shown in Table 5. The MPV mean ranks increased progressively from Stage I (n = 55, mean rank = 173.88) to Stage II (n = 100, mean rank = 232.59), to Stage III (n = 196, mean rank = 265.88), to Stage IV (n = 119, mean rank = 291.42), and to Stage V (n = 57, mean rank = 342.35). There was a statistically significant difference (H = 42.500, df = 4, p < 0.001), suggesting that MPV increases as CKD progresses from Stage 1 to Stage 5, indicating larger platelets with advancing CKD stages.

**Table 5:** Kruskal-Wallis Test Comparing MPV Across CKD Stages (N = 527) MPV = Mean Platelet Volume; CKD = Chronic Kidney Disease. Kruskal-Wallis H = 42.500, df = 4, p < 0.001. A progressive increase in MPV mean ranks was observed across CKD stages, indicating elevated MPV with advancing renal disease.

CKD Stage	N	MPV (fL), Median (Q1-Q3)	Mean Rank	Std. Deviation
Stage I	55	8.79 (8.04-9.35)	173.88	0.98
Stage II	100	9.18 (8.52-9.76)	232.59	0.95
Stage III	196	9.38 (8.77-9.84)	265.88	0.91
Stage IV	119	9.45 (8.94-10.20)	291.42	1.05
Stage V	57	9.81 (9.33-10.59)	342.35	0.93
Total	527	—	—	0.99

Table 6 presents the Spearman’s rank correlation analysis between platelet indices and bleeding outcomes among CKD patients. MPV exhibited significant positive associations with epistaxis, gum bleeding, easy bruising, prolonged bleeding, bleeding symptom score, and clinical bleeding (p < 0.05), and the highest association occurred with bleeding symptom score and clinical bleeding (r = 0.394, p < 0.001). However, the correlation between MPV and menorrhagia was not statistically significant (p = 0.052). Platelet distribution width (PDW) showed weak but significant positive correlations with prolonged bleeding, bleeding symptom score, and clinical bleeding, but not with other bleeding outcomes. In contrast, there was no significant correlation between platelet count and bleeding outcomes or symptom scores (p > 0.05).

**Table 6:** Spearman's Rank Correlation Between Platelet Indices and Bleeding Outcomes MPV = Mean Platelet Volume; PDW = Platelet Distribution Width; N = Number of participants. Spearman’s rank correlation was used to assess associations between platelet indices and bleeding outcomes. Significant p-values are indicated at p < 0.05.

Variables	Correlation Coefficient	p-Value	N
MPV and Bleeding Outcomes			
MPV vs. Epistaxis	0.231	<0.001	527
MPV vs. Gum Bleeding	0.188	<0.001	527
MPV vs. Easy Bruising	0.138	0.001	527
MPV vs. Prolonged Bleeding	0.167	<0.001	527
MPV vs. Menorrhagia	0.085	0.052	527
MPV vs. Bleeding Symptom Score	0.394	<0.001	527
MPV vs. Clinical Bleeding	0.394	<0.001	527
PDW and Bleeding Outcomes			
PDW vs. Epistaxis	-0.010	0.822	527
PDW vs. Gum Bleeding	0.074	0.088	527
PDW vs. Easy Bruising	0.023	0.604	527
PDW vs. Prolonged Bleeding	0.153	<0.001	527
PDW vs. Menorrhagia	0.081	0.063	527
PDW vs. Bleeding Symptom Score	0.131	0.003	527
PDW vs. Clinical Bleeding	0.107	0.014	527
Platelet Count and Bleeding Outcomes			
Platelet Count vs. Epistaxis	0.026	0.546	527
Platelet Count vs. Gum Bleeding	0.072	0.098	527
Platelet Count vs. Easy Bruising	-0.001	0.978	527
Platelet Count vs. Prolonged Bleeding	-0.037	0.400	527
Platelet Count vs. Menorrhagia	0.014	0.749	527
Platelet Count vs. Bleeding Symptom Score	0.019	0.671	527
Platelet Count vs. Clinical Bleeding	0.008	0.857	527

Table 7 shows Binary logistic regression analysis of factors associated with clinical bleeding of CKD patients. The overall model was statistically significant (χ² = 113.746, p < 0.001) and accounted for 26.0% of the variance for clinical bleeding. MPV was a significant predictor, with higher MPV value associated with higher odds of bleeding (OR = 2.881, p < 0.001). Lower odds of clinical bleeding were seen in males than in females (OR = 0.616; p = 0.016). There were no significant differences in age group, diabetes mellitus, and hypertension with regards to bleeding risk (p > 0.05). Overall, there was a significant association of clinical bleeding with CKD stage, with patients with CKD Stage I and Stage III having significantly higher odds of bleeding than those with CKD Stage V.

**Table 7:** Binary Logistic Regression Analysis Predicting Bleeding Symptoms (N = 527) MPV = Mean Platelet Volume; CKD = Chronic Kidney Disease; OR = Odds Ratio; CI = Confidence Interval. Reference Categories: Age Group = 18-30 years; Gender = Female; CKD Stage = Stage V, Model Statistics: Chi-square = 113.746, df = 12, p < 0.001; Nagelkerke R² = 0.260. Method = Enter (forced entry). Model fit: χ²(12) = 113.746, p < 0.001; Nagelkerke R² = 0.260; Hosmer-Lemeshow goodness-of-fit: χ²(8) = 4.599, p = 0.799; Overall classification accuracy = 69.3%. Reference category for Gender = Female; for CKD Stage = Stage V.

Variable	B	SE	Wald	df	p-Value	OR	95% CI
Mean Platelet Volume (fL)	1.058	0.126	70.893	1	<0.001	2.881	2.252-3.686
Age Group (Overall Effect)			2.365	4	0.669		
31-45 years	0.192	0.360	0.286	1	0.593	1.212	0.599-2.455
46-55 years	0.128	0.351	0.133	1	0.715	1.137	0.571-2.261
56-65 years	0.472	0.358	1.745	1	0.187	1.604	0.796-3.233
Above 65 years	0.139	0.352	0.156	1	0.693	1.149	0.577-2.291
18-30 years (Reference)	—	—	—	—	—	1.00	—
Male	-0.484	0.201	5.772	1	0.016	0.616	0.415-0.915
Female (Reference)	—	—	—	—	—	1.00	—
Diabetes Mellitus	0.324	0.200	2.610	1	0.106	1.382	0.933-2.048
Hypertension	-0.216	0.215	1.009	1	0.315	0.806	0.529-1.228
CKD Stage (Overall Effect)			15.535	4	0.004		
Stage I	1.552	0.454	11.692	1	0.001	4.719	1.939-11.485
Stage II	0.510	0.380	1.801	1	0.180	1.665	0.791-3.506
Stage III	1.016	0.346	8.596	1	0.003	2.761	1.400-5.445
Stage IV	0.661	0.366	3.268	1	0.071	1.938	0.946-3.969
Stage V (Reference)	—	—	—	—	—	1.00	—

**Figure 1:**
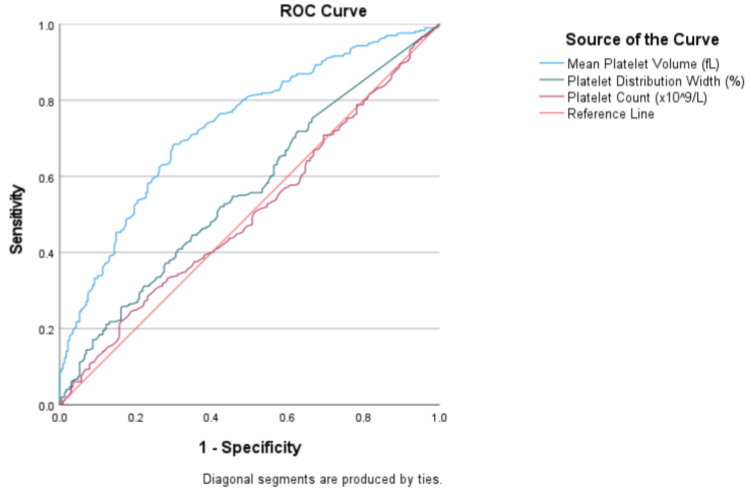
Receiver Operating Characteristic (ROC) Curves for MPV, PDW, and Platelet Count in Distinguishing Participants With and Without Bleeding Symptoms MPV = Mean Platelet Volume; PDW = Platelet Distribution Width.

Figure 1 shows the ROC curves evaluating the discriminatory ability of mean platelet volume (MPV), platelet distribution width (PDW), and platelet count for distinguishing participants with and without bleeding symptoms. MPV demonstrates the highest rate of discrimination, with its curve being the furthest from the reference line and the closest to the upper-left corner, which is more sensitive and specific. PDW is less discriminative; its curve is slightly above the reference line of the diagonal. In comparison, the platelet count curve is very close to the reference line and thus has no predictive power. To sum up, the figure shows that MPV is the most effective predictor of bleeding symptoms of the three parameters. Simultaneously, PDW is not highly informative, and platelet counts lack predictive value.

Table 8 shows the receiver operating characteristic (ROC) curve results evaluating the discriminatory ability of MPV, PDW, and platelet count for distinguishing participants with and without bleeding symptoms. The discriminatory power of MPV was acceptable, with an AUC of 0.730 (SE = 0.022, 95% CI: 0.687-0.772, p < 0.001), indicating that it could distinguish patients with bleeding from those without. The PDW test showed minimal capacity to differentiate between groups, which resulted in a statistically significant outcome (AUC = 0.562, SE = 0.025, 95% CI: 0.512-0.611, p = 0.015). The platelet count showed complete impairment in its ability to predict results, as it performed at random-chance levels (AUC = 0.505, SE = 0.025, 95% CI: 0.455-0.554, p = 0.857).

**Table 8:** ROC Curve Analysis for MPV, PDW, and Platelet Count in Relation to Bleeding Symptoms (N = 527) AUC = Area Under the Curve; SE = Standard Error; MPV = Mean Platelet Volume; PDW = Platelet Distribution Width. AUC values range from 0.5 (chance) to 1.0 (perfect discrimination). MPV demonstrated acceptable discriminatory ability (AUC = 0.730), significantly outperforming chance. PDW showed weak but statistically significant discrimination. Platelet count did not perform significantly better than chance (p = 0.857).

Predictor	AUC	SE	p	95% CI
MPV	0.730	0.022	<0.001	0.687, 0.772
PDW	0.562	0.025	0.015	0.512, 0.611
Platelet Count	0.505	0.025	0.857	0.455, 0.554

Table 9 shows the distribution of participants' symptom score categories. Mild symptoms (1-2 symptoms) were reported in most participants (n = 284, 53.9%), followed by no symptoms (n = 229, 43.5%). Severe symptoms (>3 symptoms) were observed in only 14 participants (2.7%). The results revealed that the most prevalent presentation in the study population was mild symptom burden.

**Table 9:** Bleeding Symptom Score Categories Among CKD Patients (N = 527) CKD = Chronic Kidney Disease. Symptom categories were based on total symptom scores: No Symptoms = 0 symptoms, Mild Symptoms = 1–2 symptoms, and Severe Symptoms ≥3 symptoms (N = 527).

Symptom Category	Frequency	Percent
No Symptoms	229	43.5
Mild Symptoms (1-2)	284	53.9
Severe Symptoms (>3)	14	2.7
Total	527	100.0

## Discussion

Principal findings

Our study shows a high rate of bleeding symptoms in patients with CKD, with over 50% of our participants reporting bleeding symptoms. Crucially, these changes were detected as early as the pre-dialysis stage, indicating that bleeding symptoms occur during CKD. Our study revealed a strong link between bleeding symptoms and platelet indices. MPV was significantly elevated in patients who had bleeding, and demonstrated a consistent association in regression and ROC analyses. Interestingly, platelet count did not demonstrate meaningful discriminative power, suggesting that qualitative rather than quantitative platelet measures are more important in this patient group.

Interpretation of the discordant hemostatic profile

The main finding of this study is the presence of both elevated MPV and bleeding tendency in CKD patients, consistent with a discordant hemostatic profile. An increase in MPV, commonly associated with altered platelet activity and thrombotic risk, was found to be associated with bleeding in this study. This finding is in line with reports from myeloproliferative neoplasms, in which major bleeding is associated with elevated MPV [14]. But conflicting reports from transcatheter aortic valve replacement (TAVR) cohorts have shown that low MPV is associated with bleeding in high-risk patients [15], suggesting that this effect may be context-specific.

In our study, MPV also increased with progression of CKD, consistent with previous studies showing an inverse correlation between MPV and estimated glomerular filtration rate (eGFR) and its association with systemic inflammatory processes [16,17]. These observations indicate that MPV increases, suggesting altered platelet activity as the disease progresses and may be a manifestation of inflammatory and metabolic derangements. Overall, these findings may suggest altered platelet function in CKD; however, direct platelet function testing was not performed, and therefore, this interpretation remains speculative. Altered platelet activity may be accompanied by qualitative platelet dysfunction in platelet adhesion and aggregation, resulting in poor hemostasis despite the altered platelet activity.

Qualitative platelet dysfunction may manifest as a combination of high mean platelet volume (MPV) and bleeding symptoms. In chronic kidney disease, platelet adhesion and aggregation may be less efficient due to uremic toxins, endothelial dysfunction, and chronic inflammation, even with increased platelet size. This may partially explain the coexistence of altered platelet indices and self-reported bleeding manifestations observed in this study.

Predictive role of platelet indices

MPV was significantly associated with bleeding symptoms, with higher MPV values corresponding to increased odds of reporting bleeding symptoms in the study population. This is consistent with previous reports of the independent predictive value of MPV for major bleeding and other adverse outcomes in multivariable analyses [15,18]. These findings were corroborated by Spearman’s correlation analysis, which revealed significant positive correlations between MPV and other bleeding manifestations, including bleeding symptom score and clinical bleeding. Similarly, in CKD, there are previous reports of a complex hemostatic phenotype, involving both qualitative platelet dysfunction and platelet activation and inflammation despite greater bleeding tendency [9,19]. In contrast, PDW showed only weak correlations, while platelet count was not significantly associated with bleeding outcomes in our cohort. These findings align with previous studies indicating that classical platelet indices may not fully capture bleeding risk in CKD patients [9].

Women were also at higher risk for bleeding symptoms. This finding is consistent with data from large percutaneous coronary intervention (PCI) registries and regional registries showing increased bleeding in women [20,21]. Furthermore, CKD stage was significantly associated with bleeding risk, consistent with previous reports that renal impairment leads to platelet dysfunction via inflammation or endothelial dysfunction [17,22]. Interestingly, Stage I CKD demonstrated higher odds of bleeding compared with Stage V CKD in the regression model. This unexpected finding should be interpreted cautiously and may reflect unmeasured clinical factors, differences in management, or residual confounding.

On the other hand, we found age, diabetes, and hypertension were not independently associated with bleeding. These factors have been reported to be associated with bleeding in other studies but may be weaker predictors after adjustment for stronger predictors or may differ by population [23-25]. ROC analysis also demonstrated that MPV had moderate discriminative ability for distinguishing participants with and without bleeding symptoms. In contrast, PDW showed limited utility and platelet count performed no better than chance.

Clinical implications

This study has several clinical implications. MPV may be a convenient, low-cost, and readily available biomarker associated with bleeding symptoms in patients with CKD. Moreover, bleeding and high MPV should be considered indicators that warrant individual risk assessment for the use of antiplatelet or anticoagulant agents. These results also suggest that clinical evaluation based on traditional risk factors, including platelet count, can underestimate bleeding risk and underscore the importance of using qualitative measures of platelet function.

Strengths and limitations

The present study has several strengths, including its large sample size and the consideration of clinical and laboratory measures to evaluate hemostatic function. The validity of the results is also increased with the use of various statistical approaches. Nevertheless, some limitations should be noted. The study is cross-sectional, which impedes causal inference. The findings may not be generalized because of convenience sampling and may be subject to reporting bias due to self-reported bleeding symptoms. To determine the correlation between platelet dysfunction and bleeding in CKD, longitudinal studies are needed. Medications, including antiplatelet agents and NSAIDs, could have influenced bleeding, which was not considered in the regression analysis. In addition, the bleeding assessment relied on a study-specific symptom questionnaire rather than a formally validated bleeding assessment tool, potentially affecting the precision and reproducibility of bleeding classification. Furthermore, direct platelet function testing was not performed. Therefore, the proposed explanation of qualitative platelet dysfunction remains speculative and could not be confirmed by the present study. Additionally, MPV may be influenced by pre-analytical and analytical factors, including sample processing conditions and laboratory methodology, which were not evaluated in this study.

## Conclusions

Overall, the present study suggests that chronic kidney disease (CKD) may be associated with a discordant hemostatic profile characterized by larger platelets and a higher prevalence of bleeding symptoms. A higher mean platelet volume (MPV) was strongly associated with bleeding symptoms, highlighting the importance of qualitative platelet alterations over quantitative ones (such as platelet count). These findings indicate that, rather than being a mere bleeding or thrombotic disease, hemostasis in CKD is a complex form of altered hemostasis. The clinical implications of risk evaluation and management of this dual-risk nature are important. Future research is required to elucidate the mechanisms and to assess the clinical utility of platelet indices to inform therapeutic strategies in CKD patients.

## Appendices

Appendix 1

**Table 10:** Clinical and Questionnaire-Based Assessment

Items	Responses
Age (years)	-
Gender	-
Body Mass Index (BMI)	-
Smoking Status	-
Hypertension	-
Diabetes Mellitus	-
Duration of Chronic Kidney Disease (CKD)	-
Current Medication Use	-
History of Nosebleeds (Epistaxis)	-
Gum Bleeding (e.g., while brushing)	-
Easy Bruising	-
Prolonged Bleeding from Minor Cuts (>5 minutes)	-
Heavy Menstrual Bleeding (Females only)	-
